# Characterization of KPC-287 conferring high-level ceftazidime–avibactam resistance in an ST11-KL64 *Klebsiella pneumoniae* isolate

**DOI:** 10.3389/fmicb.2026.1938185

**Published:** 2026-10-16

**Authors:** Yue Gong, Yanlei Shen, Junru Lu, Yan Li, Shangshang Qin, Xiufang Shi, Xin Tian

**Affiliations:** 1 Department of Pharmacy, The First Affiliated Hospital of Zhengzhou University, Zhengzhou, Henan, China; 2 XNA Platform, School of Pharmaceutical Sciences, Zhengzhou University, Zhengzhou, Henan, China; 3 Key Laboratory of Advanced Drug Preparation Technologies, Ministry of Education, Zhengzhou University, Zhengzhou, Henan, China; 4 School of Pharmaceutical Sciences, Zhengzhou University, Zhengzhou, China; 5 Henan Key Laboratory of Precision Clinical Pharmacy, The First Affiliated Hospital of Zhengzhou University, Zhengzhou, Henan, China

**Keywords:** ceftazidime-avibactam resistance, *Klebsiella pneumoniae*, KPC variant, KPC-287, ST11-KL64

## Abstract

**Background:**

*Klebsiella pneumoniae* is a major global public health threat because of its increasing antimicrobial resistance and capacity for dissemination. Ceftazidime-avibactam (CAZ/AVI) is a key therapeutic option for infections caused by KPC-producing *Enterobacterales*, but emerging KPC variants compromises its efficacy. Here, we characterized multidrug-resistant *K. pneumoniae* isolate KP22-83 and investigated the contribution of KPC-287 to antimicrobial resistance, bacterial fitness, and virulence.

**Methods:**

KP22-83 was characterized by antimicrobial susceptibility testing, whole-genome sequencing, resistance and virulence gene annotation, KPC phylogenetic analysis, and structural analysis. The *bla*_KPC-287_ gene was cloned into pUC19 and introduced into *Escherichia coli* DH5α and *K. pneumoniae* ATCC 13883 for functional validation. Growth, motility, *in vitro* competition, and biofilm formation assays were performed to evaluate fitness-associated phenotypes associated with KPC-287 carriage. The transferability and retention of *bla*_KPC-287_ were examined by conjugation assay and serial passage with or without CAZ/AVI. Virulence was evaluated using a *Galleria mellonella* infection model.

**Results:**

KP22-83 belonged to ST11-KL64 and exhibited multidrug-resistance. Whole-genome sequencing identified *bla*_KPC-287_ together with 11 additional antimicrobial resistance-associated genes and 34 virulence-associated genes. KPC-287 clustered close to KPC-155 and differed by P168Q and A242del while retaining a highly conserved predicted global fold. Introduction of pUC19-KPC-287 increased the CAZ/AVI MIC to 64/4 mg/L in both *E. coli* DH5α and *K. pneumoniae* ATCC 13883, demonstrating that *bla*_KPC-287_ conferred high-level CAZ/AVI resistance. In contrast, meropenem MICs were not substantially altered. KPC-287 carriage caused modest reductions in growth, motility, and competitive fitness, together with greater crystal violet-stained biofilm biomass than the corresponding empty-vector controls. The *bla*_KPC-287_ was detected in transconjugants together with IncFII and IncR replicon markers and remained detectable after 30 passages with or without CAZ/AVI. Furthermore, KP22-83 caused substantial larval mortality, whereas KPC-287 acquisition did not markedly affect virulence in *K. pneumoniae* ATCC 13883.

**Conclusion:**

This study provides a systematic functional characterization of KPC-287 in an ST11-KL64 *K. pneumoniae* clinical isolate in China. KPC-287 confers high-level CAZ/AVI resistance with limited fitness cost and *bla*_KPC-287_ is transferable in an IncFII/IncR plasmid background and remains detectable during serial passage. These findings highlight the need for continuous genomic surveillance and functional characterization of emerging KPC variants.

## 1 Introduction

*Klebsiella pneumoniae* is a prominent opportunistic Gram-negative pathogen that can cause a broad spectrum of healthcare-associated and community-acquired infections, including pneumonia, bloodstream infections, urinary tract infections, and liver abscess, particularly in immunocompromised individuals (Lei et al., 2024; Heng et al., 2025). The global clinical burden posed by *K. pneumoniae* has increased with the dissemination of carbapenem-resistant strains and the emergence of lineages in which antimicrobial resistance and hypervirulence have converged (Pu et al., 2023; Lei et al., 2024). The 2024 World Health Organization Bacterial Priority Pathogens List ranked carbapenem-resistant *K. pneumoniae* (CRKP) as the highest-priority bacterial threat (Sati et al., 2025). Carbapenem resistance in *K. pneumoniae* is frequently mediated by acquired carbapenemases, particularly KPC-type class A serine carbapenemases (Brunetti et al., 2026). The *bla*_KPC_ genes are commonly carried on mobile plasmids, enabling horizontal transmission across bacterial hosts (Heng et al., 2025).

Ceftazidime-avibactam (CAZ/AVI), which combines the third-generation cephalosporin ceftazidime with the diazabicyclooctane β-lactamase inhibitor avibactam, has become an important therapeutic option for infections caused by KPC-producing Enterobacterales (Di Bella et al., 2021). However, increasing reports of CAZ/AVI-resistant KPC-producing isolates have raised concerns regarding the long-term clinical utility of this agent (Di Bella et al., 2021). CAZ/AVI resistance can arise through multiple mechanisms, including increased *bla*_KPC_ expression or copy number, altered outer-membrane permeability, changes in efflux activity, and structural variation in KPC enzymes (Tumbarello et al., 2021; Xie et al., 2025). Among these, KPC variation is one of the most extensively documented mechanisms in KPC-producing isolates (Di Bella et al., 2021; Li et al., 2026). The KPC family now comprises more than 270 assigned variants, and CAZ/AVI resistance-associated changes are concentrated primarily within the 164–179 omega loop, the 237–243 loop, and the 267–275 loop (Brunetti et al., 2026). Recent studies of KPC-275 and KPC-271 demonstrated the phenotypic diversity of KPC variation, as both conferred high-level CAZ/AVI resistance but differ in their effects on carbapenem susceptibility and bacterial fitness (Huang et al., 2026; Ou et al., 2026). Therefore, the clinical significance of individual KPC variants should be established through experimental validation rather than inference from sequence changes alone.

In this study, we characterized KP22-83, a multidrug-resistant ST11-KL64 *K. pneumoniae* clinical isolate carrying *bla*_KPC-287_. Genomic and functional analyses were performed to determine the contribution of KPC-287 to CAZ/AVI resistance and carbapenem susceptibility. We further evaluated fitness-associated phenotypes, plasmid transferability and retention, and virulence associated with KPC-287 carriage.

## 2 Materials and methods

### 2.1 Sample collection

*K. pneumoniae* strain KP22-83 was a clinical isolate recovered from bronchoalveolar lavage fluid on January 20, 2022. *Escherichia coli* DH5α and *K. pneumoniae* ATCC 13883 were used as recipient backgrounds for cloning-based functional validation. The laboratory-preserved hygromycin-resistant recipient strain YZ6 was employed as the recipient in conjugation assays. *E. coli* ATCC 25922 was used as the quality control strain for antimicrobial susceptibility testing. Unless otherwise stated, bacteria were cultured in Luria-Bertani (LB) broth (Aoboxing Biotech, Beijing, China) or on LB agar at 37 °C.

### 2.2 Antimicrobial susceptibility testing

Antimicrobial susceptibility testing was performed using the broth microdilution method according to the Clinical and Laboratory Standards Institute (CLSI) guidelines. The antibiotics tested included meropenem, ceftazidime, ceftazidime-avibactam (CAZ/AVI; avibactam fixed at 4 mg/L), aztreonam, amikacin, tobramycin, ciprofloxacin, doxycycline, tigecycline, and polymyxin B. Briefly, a single colony was inoculated into Mueller-Hinton broth (MHB; Hope Bio-Technology, Qingdao, China) and cultured overnight at 37 °C with shaking. The overnight culture was diluted into fresh MHB and grown to the logarithmic phase. Twofold serial dilutions of the antibiotics were prepared in 96-well plates and cultured with bacterial suspensions. Following incubation at 37 °C for 18 h, bacterial growth was evaluated to determine the minimum inhibitory concentrations (MICs). All assays were performed in at least three independent experiments, with *E. coli* ATCC 25922 included as the quality-control strain. Susceptibility categories were interpreted according to CLSI M100 guidelines (35th edition, 2025), except for tigecycline, which was interpreted based on EUCAST.

### 2.3 Whole-genome sequencing and bioinformatics analysis

Genomic DNA from KP22-83 was extracted using the TIANamp Genomic DNA Kit (Tiangen Biotech, Beijing, China), and DNA concentration was measured using a Qubit 4 Fluorometer (Thermo Fisher Scientific, Waltham, MA, USA). Whole-genome sequencing (WGS) was performed on the Illumina HiSeq 2,500 platform to generate 150-bp paired-end reads, which were subsequently assembled using SPAdes v3.14.0. Multilocus sequence typing was performed using mlst v2.30.0. Species assignment, capsular locus, and related *Klebsiella* genomic features were assessed using Kleborate v2.1.0 (Lam et al., 2021). Antimicrobial resistance genes were identified using ResFinder v4.6.0 (Bortolaia et al., 2020; Carattoli and Hasman, 2020). Virulence-associated genes were screened against the Virulence Factor Database (VFDB) core dataset (Kleinheinz et al., 2014), and insertion sequences were annotated using ISfinder (Siguier et al., 2006). Genome annotation was performed using the RAST server, followed by manual curation. BLASTn searches against the NCBI nucleotide database were used to confirm the identities of selected genes. KPC protein sequences available in the NCBI database as of May 8, 2026, were retrieved and aligned using Clustal Omega v1.2.2. The phylogenetic tree was constructed in MEGA and visualized using iTOL^1^ (Letunic and Bork, 2021).

Plasmid replicons were screened using PlasmidFinder v2.0.1. Draft assemblies of the remaining isolates generated from short-read sequencing were aligned against these reference plasmids using BLASTn. A putative plasmid-background assignment was made when the matched region showed both nucleotide identity and sequence coverage greater than 80%. Brig v.0.95 was used to generate plasmid comparison figures.

### 2.4 Sequence and structural comparison of KPC-287 and KPC-155

The nucleotide and amino acid sequences of *bla*_KPC-155_/*bla*_KPC-287_ and KPC-155/KPC-287, respectively, were aligned by ClustalW^2^ and visualized using ESPript 3.0.^3^

Predicted structures of KPC-155 and KPC-287 variant were generated using AlphaFold Server^4^ (Abramson et al., 2024). The structures were visualized and superimposed in PyMOL v.3.1.1, and the root-mean-square deviation (RMSD) was calculated during structural superposition.

### 2.5 Cloning experiments

The DNA fragment that contained the native upstream promoter and open reading frame (ORF) of *bla*_KPC-287_ was amplified from KP22-83 genomic DNA and ligated to vector pUC19-Apr using C117 one-step cloning enzyme (Vazyme, Nanjing, China) to generate pUC19-KPC-287. Primers used for plasmid construction and verification are listed in Supplementary Table S1. The ligation product was transformed into chemically competent *E. coli* DH5α by heat shock, and into electrocompetent *K. pneumoniae* ATCC 13883 by electroporation at 2,000 V, 25 μF, and 200 Ω. Positive transformants were selected on LB agar containing 10 μg/mL apramycin, and verified by colony PCR and DNA sequencing. The empty-vector controls were designated DH5α-pUC19 and ATCC 13883-pUC19, while the recombinant strains were designated DH5α-pUC19-KPC-287 and ATCC 13883-pUC19-KPC-287, respectively.

### 2.6 Growth curve assay

Single colonies of the tested strains were inoculated into 1 mL LB broth and incubated overnight at 37 °C with shaking at 200 rpm. The overnight cultures were diluted 1:100 into fresh LB broth and further incubated under the same conditions for 24 h. Bacterial growth was monitored by measuring OD_600_ every 2 h using a microplate reader (Flash Spectrum Biological Technology, Shanghai, China), with all experiments performed in triplicate.

### 2.7 Motility assay

Single colonies of the tested strains were inoculated into 1 mL LB broth and incubated overnight at 37 °C with shaking at 200 rpm. A 5-μL aliquot of each suspension was inoculated into the center of a semisolid motility plate (0.3% agar). After static incubation at 37 °C for 36 h, the migration diameters were measured using calipers. The assay was conducted in at least three independent experiments.

### 2.8 *In vitro* competition assay

*In vitro* competition assays were performed between DH5α-pUC19 and DH5α-pUC19- KPC-287, as well as between ATCC 13883-pUC19 and ATCC 13883-pUC19-KPC-287. Single colonies were inoculated into 1 mL LB broth and cultured overnight at 37 °C with shaking at 200 rpm. The bacterial cultures were normalized to a 0.5 McFarland standard and mixed at a 1:1 ratio. The initial mixtures (0 h) and subsequent cultures obtained during serial passaging (10 μL transferred into 1 mL fresh LB every 24 h up to 72 h) were enumerated on nonselective LB agar and selective LB agar containing CAZ/AVI at 8/4 μg/mL. The competition index (W) was calculated using the formula: *W* = ln (*R*_F_/*R*_I_)/ln (*S*_F_/*S*_I_), where R and S represent the CFU counts of the KPC-287-carrying and empty-vector strains, respectively, at the initial (I) and final (F) time points. *W* = 1, *W* > 1, and *W* < 1 indicated neutral, advantageous, and disadvantageous competitive fitness for the KPC-287-carrying strain, respectively. The experiment was performed in triplicate.

### 2.9 Biofilm formation assay

Biofilm formation was quantified using a standard crystal violet microtiter plate assay. Single colonies were inoculated into 2 mL tryptic soy broth (TSB; Aoboxing Biotech, Beijing, China) and cultured overnight at 37 °C with shaking at 200 rpm. Overnight cultures adjusted to a 0.5 McFarland standard were transferred into sterile 96-well plates (200 μL/well), and incubated statically at 37 °C for 72 h. Subsequently, the culture supernatant containing planktonic cells was aspirated, and the wells were washed thrice with phosphate-buffered saline (PBS; Biosharp, Beijing, China) to remove non-adherent bacteria. Biofilms were fixed with 200 uL methanol for 15 min, air dried, and stained with 100 μL 0.1% crystal violet (Zhiyuan, Tianjin, China) for 10 min. After excess dye was removed by washing with PBS, the biofilm-bound dye was dissolved in 100 μL 30% acetic acid for 30 min. Absorbance was measured at 590 nm using a microplate reader (Flash Spectrum Biological Technology, Shanghai, China).

### 2.10 Conjugation assay

Conjugation assays were performed using KP22-83 as the donor and the laboratory-preserved hygromycin-resistant strain YZ6 as the recipient. Log-phase donor and recipient cultures (OD_600_ ≈ 0.5) were mixed at a 1:1 ratio, and 100 μL of the mixture was spotted onto antibiotic-free LB agar for overnight mating at 37 °C. The resulting bacterial lawn was harvested in 1 mL of sterile saline, and plated onto LB agar containing hygromycin (100 μg/mL) and CAZ/AVI (16/4 μg/mL). The resulting transconjugants were verified by PCR targeting *bla*_KPC-287_ and the IncFII and IncR replicon markers. The assay was conducted in triplicate as three independent biological replicates. This assay was designed to confirm plasmid transferability qualitatively.

### 2.11 Plasmid stability assay

Stability of *bla*_KPC-287_ in KP22-83 was assessed by serial passage with or without CAZ/AVI selective pressure (32/4 μg/mL). For each condition, a single colony was inoculated into 1 mL fresh LB broth and cultured at 37 °C with shaking at 200 rpm for 12 h (passage 0, P0), Cultures were diluted 1:100 and passaged every 12 h for 30 passages (P30). The P30 cultures were then plated onto antibiotic-free MacConkey agar, and 96 randomly selected colonies per condition were analyzed by colony PCR targeting *bla*_KPC-287_.

### 2.12 *Galleria mellonella* infection model

Active, non-melanized *Galleria mellonella* larvae (25 ± 5 mm, 300 ± 50 mg; Keyun, Jiyuan, China) were acclimated at 37 °C for 30 min. Ten larvae per group were injected with 10 μL of log-phase bacterial suspension (10^3^ CFU/larva, verified via plate counting). Following static incubation at 37 °C for 72 h, and survival was recorded every 12 h, with death defined by complete melanization and failed to respond to stimulation. Survival curves were generated and analyzed using GraphPad Prism v10.1.2.

### 2.13 Statistical analysis

Statistical analyses were performed using GraphPad Prism v10.1.2. Data are presented as the mean ± standard deviation from at least three independent experiments. Statistical significance was determined by Student’s *t*-test. *p* value < 0.05 was considered statistically significant. Significance levels are indicated as ^*^p < 0.05, ^**^p < 0.01, and ^***^p < 0.001.

### 2.14 Data availability

Genome sequence data reported i-n this study have been deposited in the National Center for Biotechnology Information under BioProject accession no. PRJNA1494798.

## 3 Results

### 3.1 Isolation and characterization of the *K. pneumoniae* clinical isolate KP22-83

The clinical isolate designated KP22-83 was recovered on January 20, 2022, from bronchoalveolar lavage fluid collected from a 58-year-old male patient admitted to the comprehensive ICU. Whole-genome analysis identified KP22-83 as an ST11-KL64 *K. pneumoniae* strain. Antimicrobial susceptibility testing showed that KP22-83 exhibited a multidrug-resistant phenotype, with high MICs to meropenem, ceftazidime, aztreonam, doxycycline, tobramycin, amikacin, and ciprofloxacin, while retaining a low MIC to polymyxin B (Table 1). Genomic analysis identified 12 antimicrobial resistance-associated genes: *aadA2*, *bla*_KPC-287_, *bla*_LAP-2_, *bla*_SHV-12_, *bla*_TEM-1_, *catA2*, *dfrA14*, *fosA6*, *qnrS1*, *rmtB1*, *sul2*, and *tet(A)*. In addition, KP22-83 harbored 34 virulence-associated genes, predominantly involved in siderophore-mediated iron acquisition and bacterial adherence. These included the enterobactin biosynthesis and transport genes *entA*, *entB*, *entE*, *entS*, *fepA*, *fepB*, *fepC*, *fepD*, and *fepG*; the yersiniabactin-associated genes *fyuA*, *irp1*, *irp2*, *ybtA*, *ybtE*, *ybtP*, *ybtQ*, *ybtS*, *ybtT*, *ybtU*, and *ybtX*; the aerobactin-associated genes *iucA*, *iucB*, *iucC*, *iucD*, and *iutA*; and the adherence-associated genes *fimA*, *fimE*, *ompA*, *ecpA*, *ecpB*, *ecpC*, *ecpD*, *ecpE*, and *ecpR*. Collectively, these findings characterized KP22-83 as a clinically important multidrug-resistant ST11-KL64 *K. pneumoniae* isolate and warranted further investigation into the contribution of KPC-287 to its resistance phenotype.

**Table 1:** Antibiotic susceptibility of *K. pneumoniae* KP22-83.

Strains	MEM	TGC	PMB	CAZ	ATM	DO	TOB	AMK	CIP	CAZ/AVI	FEP/ZID
KP22-83	256	2	<0.125	≥64.0	≥64.0	≥16.0	≥16.0	≥64.0	≥4.0	640/4	16/4

MEM, meropenem; TGC, tigecycline; PMB, polymyxin B; CAZ, ceftazidime; ATM, aztreonam; DO, doxycycline; TOB, tobramycin; AMK, amikacin; CIP, ciprofloxacin; CAZ/AVI, ceftazidime-avibactam, avibactam was tested at a fixed concentration of 4 mg/L; FEP/ZID, cefepime-zidebactam, zidebactam was tested at a fixed concentration of 4 mg/L.

### 3.2 KPC-287 clustered close to KPC-155 with local structural differences

The *bla*_KPC-287_ identified in KP22-83 showed 100% identity at nucleotide and amino-acid levels to the previously deposited KPC-287 sequence from *K. pneumoniae* strain 15,143 (GenBank accession PX776553.1). The corresponding record was derived from a clinical isolate recovered from sputum in China. However, detailed experimental characterization of KPC-287, including its contribution to antimicrobial susceptibility and associated biological phenotypes, has not been reported to date. To determine the phylogenetic placement of KPC-287 within the KPC family, KPC protein sequences available as of May 8, 2026, were aligned and subjected to phylogenetic analysis. KPC-287 clustered close to KPC-155, indicating a close sequence relationship between these two variants (Figure 1A). Nucleotide and amino-acid sequence alignment showed that KPC-287 and KPC-155 were highly conserved overall (Figure 1B; Supplementary Figure S1). However, KPC-287 differed from KPC-155 by two major changes: a proline-to-glutamine substitution at residue 168 (P168Q) and deletion of alanine at residue 242 (A242del), corresponding to one single-nucleotide substitution and one three-nucleotide deletion, respectively (Figure 1B; Supplementary Figure S1).

**Figure 1:**
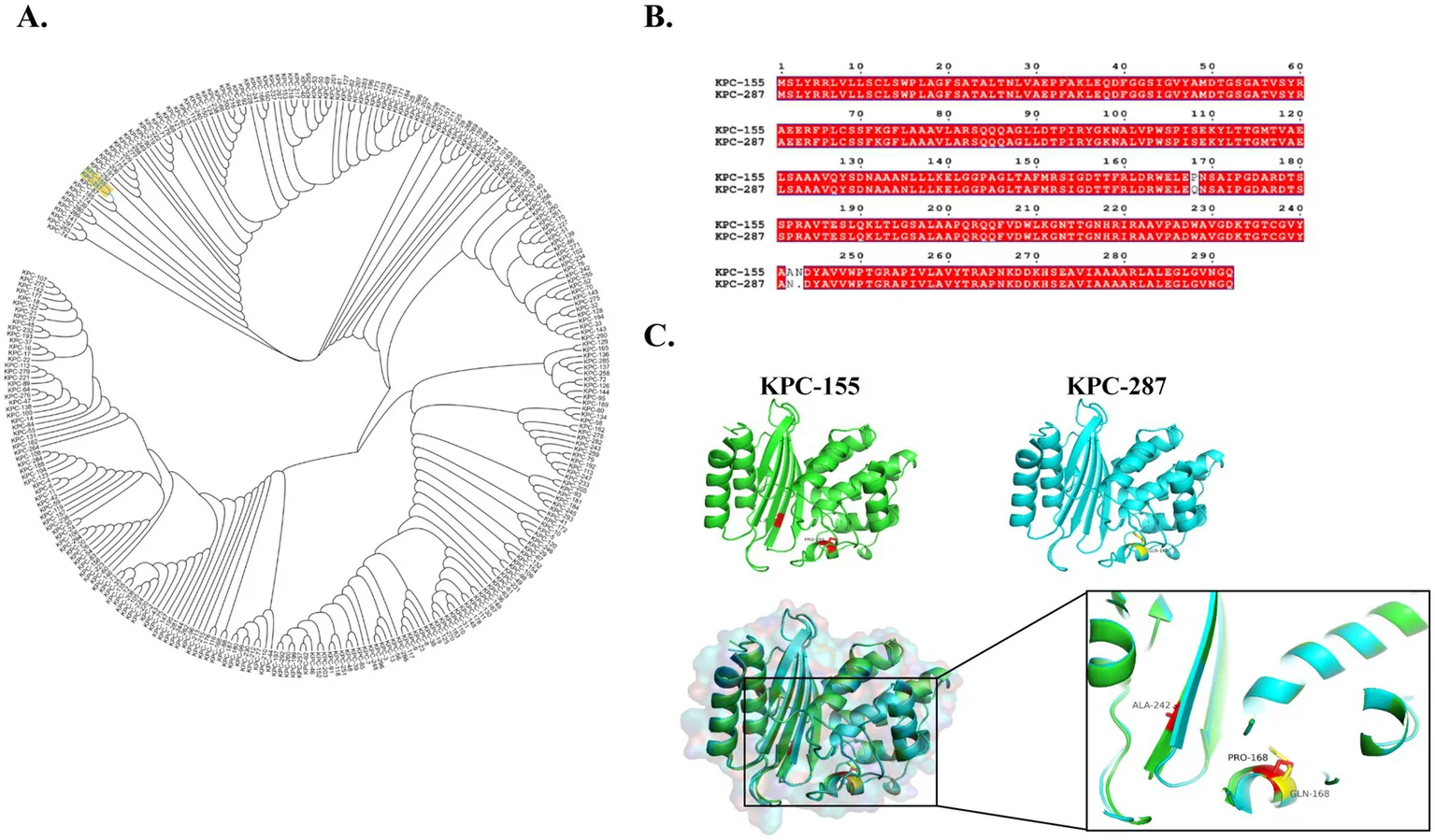
Phylogenetic, sequence, and predicted structural analysis of KPC-287. **(A)** Phylogenetic tree of KPC variants showing the placement of KPC-287 within the KPC family. **(B)** Amino acid sequences of KPC-287 and KPC-155 were aligned using ClustalW and presented using ESPript 3.0. (C) AlphaFold-predicted structures of KPC-155 and KPC-287 were visualized and superimposed using PyMOL.

To assess whether these changes were associated with structural alterations, AlphaFold-predicted models of KPC-155 and KPC-287 were superimposed using PyMOL. Both variants retained the typical KPC β-lactamase architecture and showed a highly similar global fold, with an RMSD of 0.093 (Figure 1C). The main differences were localized near residue 168 and the Ala242 deletion site. The P168Q substitution may alter local side-chain properties and flexibility, whereas A242del may affect the arrangement of the adjacent β-strand/loop region. However, these predicted structural differences do not establish the individual contribution of each alteration to the CAZ/AVI-resistance phenotype. Together, these findings indicate that KPC-287 is closely related to KPC-155 but contains localized sequence and predicted structural differences, warranting functional validation of its resistance phenotype.

### 3.3 KPC-287 conferred high-level ceftazidime-avibactam resistance

To determine the contribution of KPC-287 to the resistance phenotype, *bla*_KPC-287_ was cloned into pUC19 and introduced into *E. coli* DH5α and *K. pneumoniae* ATCC 13883. Introduction of pUC19-KPC-287 increased the CAZ/AVI MIC from <0.25/4 to 64/4 mg/L in both *E. coli* DH5α and *K. pneumoniae* ATCC 13883 backgrounds, converting both recipient strains from susceptible to resistant (Table 2). In contrast, the meropenem MICs remained unchanged after acquisition of pUC19-KPC-287 in both DH5α and ATCC 13883. The empty vector had no detectable effect on the susceptibility of either strain.

**Table 2:** Antimicrobial susceptibility profiles of *bla*_KPC-287_ recombinant strains and corresponding controls.

Strains	MIC (mg/L)	
	MEM	CAZ/AVI
DH5α	<0.0625	<0.25/4
DH5α-pUC19	<0.0625	<0.25/4
DH5α-pUC19-KPC-287	<0.0625	64/4
ATCC 13883	0.0625	<0.25/4
ATCC 13883-pUC19	0.0625	<0.25/4
ATCC 13883-pUC19-KPC-287	0.0625	64/4
ATCC 25922	<0.0625	<0.25/4

MIC, minimum inhibitory concentrations; MEM, meropenem; CAZ/AVI, ceftazidime-avibactam, avibactam was tested at a fixed concentration of 4 mg/L; S, susceptible; R, resistant.

Together, these results establish KPC-287 as a determinant of high-level CAZ/AVI resistance in both *E. coli* and *K. pneumoniae*, whereas its expression had no measurable effect on meropenem susceptibility.

### 3.4 KPC-287 expression was associated with modest fitness cost

To evaluate the potential fitness effects associated with KPC-287 expression, growth, motility, *in vitro* competition, and biofilm formation assays were performed using *E. coli* DH5α and ATCC 13883-derived strains carrying either the empty pUC19 vector or pUC19-KPC-287. Growth curves were broadly similar among the tested strains. However, the pUC19-KPC-287 transformant exhibited slightly lower OD_600_ values in both DH5α and ATCC 13883 background, indicating that KPC-287 expression imposed a measurable but limited impairment of bacterial growth (Figure 2A). In contrast, pUC19-KPC-287 carriage significantly reduced motility in both bacterial backgrounds, as indicated by smaller migration diameters after 36 h of incubation on 0.3% agar (Figure 2B). *In vitro* competition assays showed that the relative fitness values remained below 1 and decreased over time in both DH5α and ATCC 13883 backgrounds (Figure 2C). Conversely, the pUC19-KPC-287 transformants produced significantly greater crystal violet-stained attached biomass than the corresponding empty-vector controls in both bacterial backgrounds (Figure 2D).

**Figure 2:**
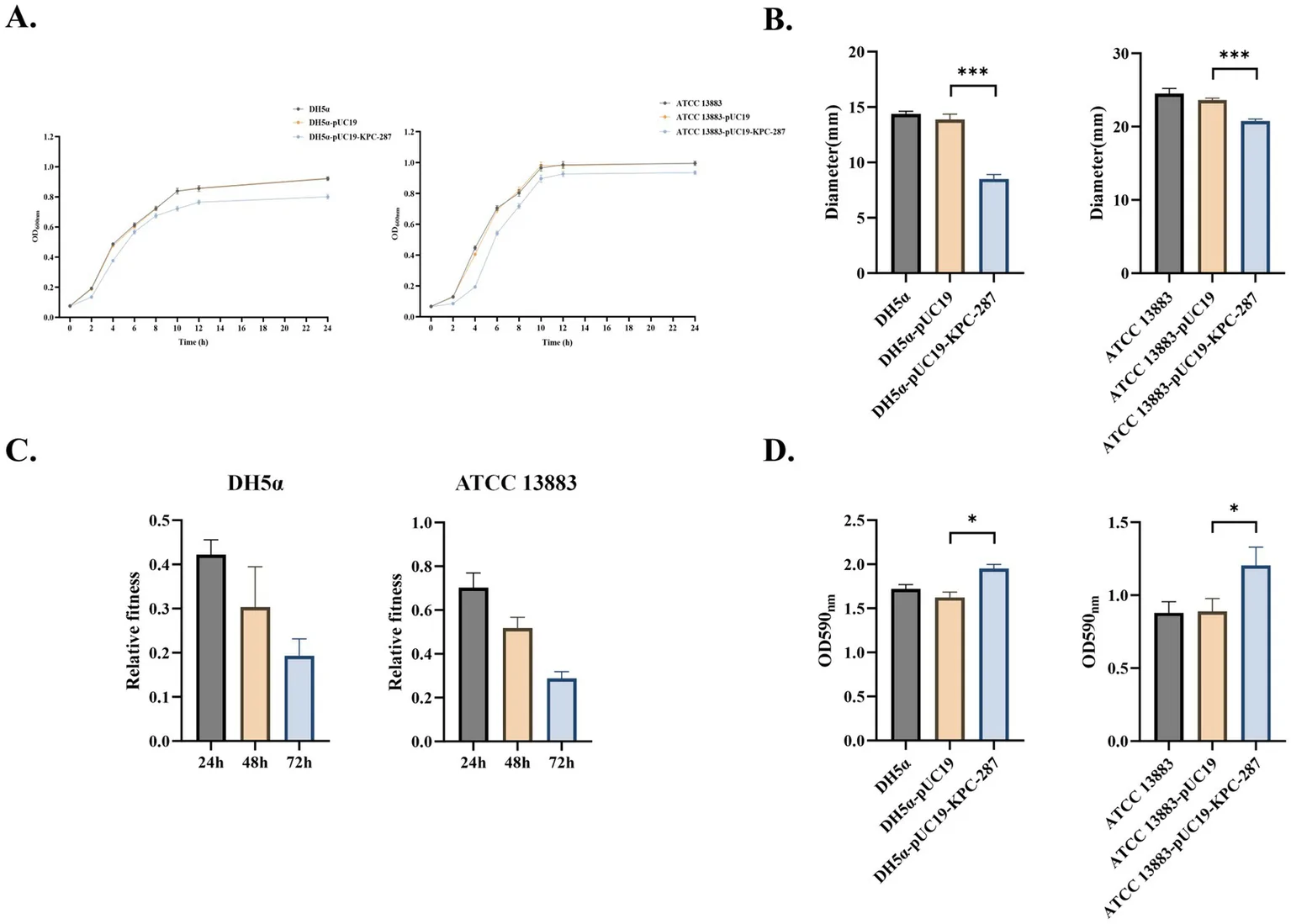
Fitness-associated phenotypes of KPC-287 in *E. coli* DH5α and *K. pneumoniae* ATCC 13883. **(A)** Growth curves of DH5α and ATCC 13883 recombinant strains. **(B)** Motility of the corresponding strains on semisolid agar. **(C)** Competitive fitness of KPC-287-producing strains relative to empty-vector controls. **(D)** Attached biofilm biomass quantified by crystal violet staining. Data are presented as the mean ± standard deviation. Statistical significance was determined by Student’s *t*-test. ^*^p < 0.05; ^***^p < 0.001.

Together, the recombinant pUC19-KPC-287 constructs showed modest growth, motility, and competitive disadvantages and produced greater crystal violet-stained attached biomass than the empty-vector controls. Because no construct carrying an ancestral or alternative KPC allele was included, the biofilm-associated difference should not be interpreted as a KPC-287 variant-specific effect.

### 3.5 Transferability and retention of *bla*_KPC-287_ in the IncFII/IncR plasmid background

Sequence analysis showed that *bla*_KPC-287_ was located on a hybrid IncFII_pHN7A8_: IncR plasmid (Figure 3). The *bla*_KPC-287_ gene was embedded within an insertion sequence-rich region adjacent to IS*Kpn27*, IS*Swi1*, and IS*26*. The presence of multiple IS*26* copies throughout the plasmid indicated a mosaic multidrug-resistance plasmid architecture with potential for horizontal dissemination and structural rearrangement.

**Figure 3:**
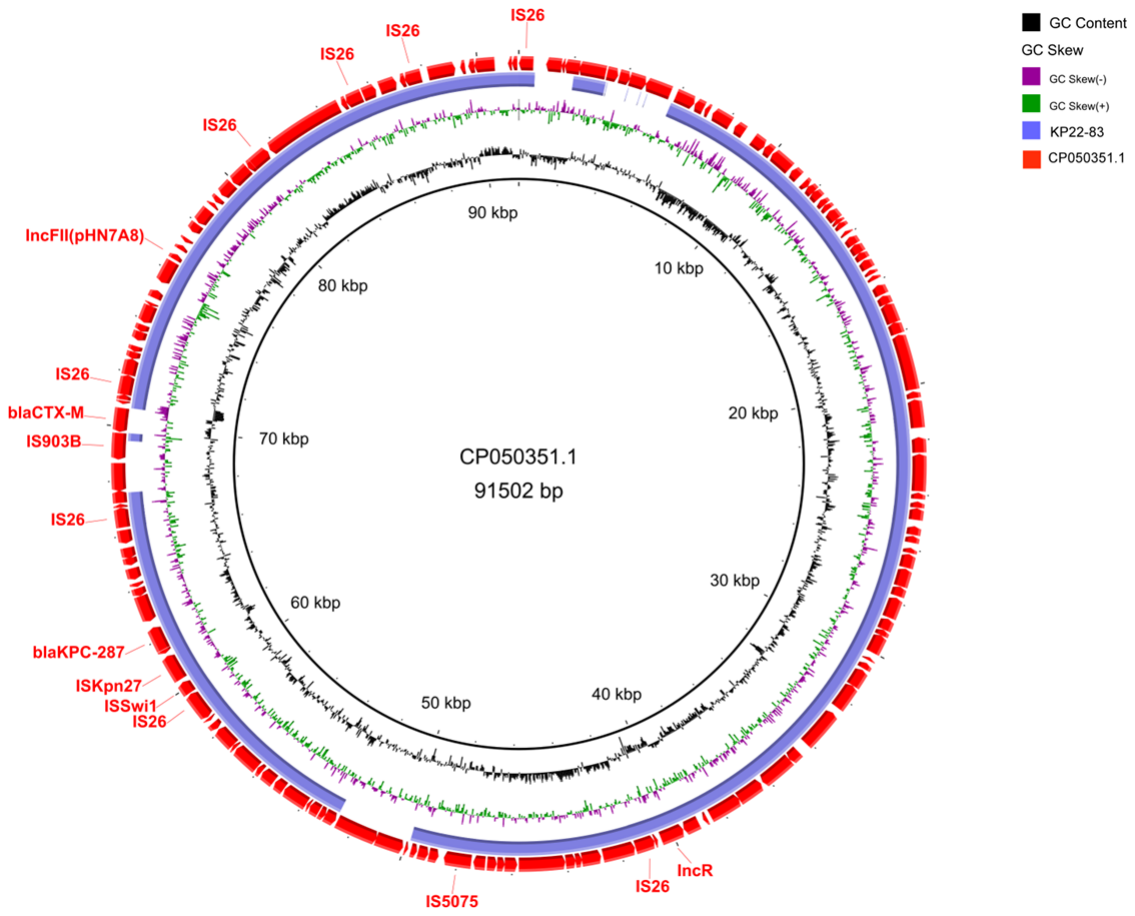
Genetic context of the *bla*_KPC-287_-bearing hybrid IncFII_pHN7A8_: IncR plasmid in KP22-83. Circular map of the *bla*_KPC-287_-bearing IncFII_pHN7A8_: IncR plasmid CP050351.1 identified in KP22-83. The map shows plasmid size, GC content, GC skew, insertion sequences, and plasmid replicons. The *bla*_KPC-287_ gene was located in an insertion-sequence-rich region adjacent to IS*Kpn27*, IS*Swi1*, and IS*26*.

Following mating of KP22-83 with YZ6, transconjugants carrying *bla*_KPC-287_ and together with IncFII and IncR replicon markers were identified, supporting the transferability of *bla*_KPC-287_ in an IncFII/IncR plasmid background under the tested mating conditions. After 30 consecutive passages, *bla*_KPC-287_ remained detectable under both antibiotic-free conditions and subinhibitory CAZ/AVI exposure.

### 3.6 KPC-287 expression did not enhance virulence-associated phenotype in the ATCC 13883 background

To evaluate whether expression of KPC-287 alone influenced virulence-associated phenotypes, *Galleria mellonella* larvae were infected with *K. pneumoniae* ATCC 13883, ATCC 13883-pUC19, ATCC 13883-pUC19-KPC-287, KP22-83, or the hypervirulent control strain KP19-2065. KP19-2065 caused the greatest larval mortality, with 40% survival at 72 h post-infection (Figure 4). Although KP22-83 was less virulent than KP19-2065, it also caused substantial larval mortality, with 50% survival at 72 h. In contrast, survival was comparable among larvae infected with ATCC 13883, ATCC 13883-pUC19, and ATCC 13883-pUC19-KPC-287. These results indicate that expression of KPC-287 did not measurably enhance larval killing in the ATCC 13883 background. The pronounced virulence observed in KP22-83 is likely influenced by multiple virulence-associated determinants within its broader genetic background.

**Figure 4:**
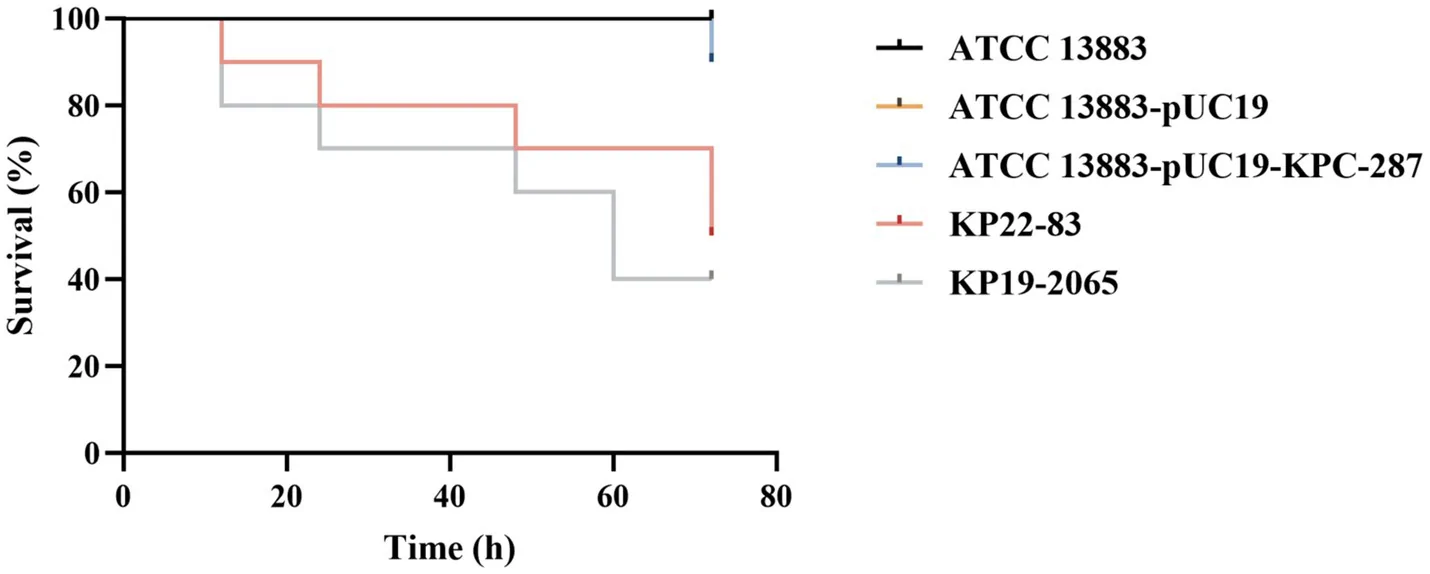
Virulence assessment of KP22-83 and KPC-287-producing strains in the *Galleria mellonella* infection model. Survival curves of *G. mellonella* larvae infected with *K. pneumoniae* ATCC 13883, ATCC 13883-pUC19, ATCC 13883-pUC19-KPC-287, KP22-83, or the hypervirulent control strain KP19-2065. Larvae were infected with 10^3^ CFU/larve and survival was monitored for 72 h.

## 4 Discussion

This study provides the first functional evidence that KPC-287 confers high-level CAZ/AVI resistance while preserving meropenem susceptibility. Importantly, *bla*_KPC-287_ was associated with a transferable IncFII/IncR-related genetic element and showed limited fitness-associated effects in recombinant hosts.

The P168Q substitution and A242 deletion in KPC-287 occur within two regions frequently implicated in the evolution of CAZ/AVI resistance: the 164–179 Ω-loop and the 237–243 loop (Brunetti et al., 2026). Structural studies have established that changes within the Ω-loop can weaken stabilizing interactions and increase conformational flexibility, potentially improving accommodation of the ceftazidime side chain and altering avibactam susceptibility (Alsenani et al., 2022). KPC-31, a D179Y derivative of KPC-3, was the first clinically described KPC variant associated with CAZ/AVI resistance together with restoration of meropenem susceptibility, establishing the characteristic phenotype subsequently reported for several Ω-loop variants (Shields et al., 2017; Haidar et al., 2017). The stepwise acquisition of D179Y and T243P in KPC-275 further illustrates how changes in the Ω and 240 loops can collectively enhance ceftazidime hydrolysis and reduce susceptibility to avibactam (Huang et al., 2026). In the present study, structural superposition of KPC-287 and KPC-155 yielded a low RMSD of 0.093, indicating preservation of the overall β-lactamase fold, with localized differences near residues 168 and 242. Given their location within CAZ/AVI resistance-associated regions, P168Q and A242del may contribute to the CAZ/AVI-resistance phenotype of KPC-287. However, the present study does not determine the individual contribution of each alteration, and their specific roles in mediating CAZ/AVI resistance remain to be further elucidated.

The cloning experiments provide direct evidence that KPC-287 is sufficient to confer high-level CAZ/AVI resistance in both *E. coli* DH5α and *K. pneumoniae* ATCC 13883 backgrounds. However, because the functional validation was performed using a high-copy-number pUC19 vector, the observed resistance phenotype may be influenced by gene expression level and may not completely reflect its effect in the native IncFII/IncR plasmid context. Despite the marked increase in CAZ/AVI MIC, KPC-287 expression did not increase the meropenem MIC in either host. Previous studies have likewise demonstrated substantial variability in carbapenem susceptibility among CAZ/AVI-resistant KPC variants (Zhang et al., 2024; Liu et al., 2025; Huang et al., 2026; Ou et al., 2026).

In the recombinant systems used here, pUC19-KPC-287 was associated with modest growth, motility, and competitive disadvantages, whereas crystal violet staining showed greater attached biomass than in the empty-vector controls. Similar heterogeneity has been reported for other CAZ/AVI-resistant KPC variants. KPC-189 showed no detectable fitness cost, whereas KPC-271 and KPC-275 were associated with impaired fitness (Zhang et al., 2024; Huang et al., 2026; Ou et al., 2026). These observations suggest that the fitness consequences of KPC variation depend on the specific variant and bacterial background. However, greater crystal violet staining describes a phenotype of the tested recombinant constructs and does not establish that the KPC-287 variant directly regulates biofilm formation. Although biofilm-associated changes have been observed in CAZ/AVI-resistant KPC-producing *K. pneumoniae* under antibiotic selection (Li et al., 2026), evidence for a KPC variant-specific causal mechanism remains limited. Because KPC-287 was not compared with KPC-2, KPC-155, or another KPC allele in the same vector and host, the observed difference could reflect recombinant expression level, cloning context, host background, or indirect physiological effects. Isogenic allele comparisons and mechanistic validation are required to determine whether KPC-287 specifically contributes to biofilm development.

KP22-83 harbored a *bla*_KPC-287_-bearing IncFIIpHN7A8: IncR plasmid with multiple insertion sequences, including IS*Kpn27*, IS*Swi1*, and IS*26* adjacent to *bla*_KPC-287_. This insertion sequence-rich organization may facilitate genetic rearrangement and horizontal dissemination (Ikhimiukor et al., 2024). In conjugation experiments, *bla*_KPC-287_ was detected in the transconjugants together with the IncFII and IncR replicon markers, supporting its transferability in an IncFII/IncR plasmid background under the tested conditions. However, PCR-based detection cannot exclude structural rearrangements of the plasmid during transfer.

KP22-83 caused substantial larval mortality, whereas the KPC-287-producing ATCC 13883 transformant showed survival comparable to the parental and empty-vector strains. These findings indicate that KPC-287 did not independently enhance virulence in the tested ATCC 13883 background. The virulence of KP22-83 is more likely related to its additional virulence-associated genes, including aerobactin, yersiniabactin, enterobactin, fimbrial, and adherence-associated determinants.

Several limitations should be acknowledged. First, cloning in pUC19 may not reflect the native *bla*_KPC-287_ expression, and functional validation was limited to two laboratory hosts. Only one clinical isolate was examined in this study. Therefore, the prevalence, evolutionary origin, and geographic distribution of KPC-287 remain unknown. In addition, the *G. mellonella* infection model is useful for preliminary virulence assessment but cannot substitute for mammalian infection models. Biofilm was evaluated only by crystal violet staining, and no ancestral or alternative KPC allele was tested in the same vector-host background; therefore, a KPC-287-specific effect on biofilm formation cannot be inferred.

## 5 Conclusion

This study functionally characterized KPC-287 in KP22-83, a multidrug-resistant ST11-KL64 *K. pneumoniae* clinical isolate. KPC-287 conferred high-level CAZ/AVI resistance in both *E. coli* and *K. pneumoniae* without increasing the meropenem MIC. Expression of KPC-287 in recombinant hosts was associated with modest fitness effects, and *bla*_KPC-287_ showed transferability in an IncFII/IncR plasmid background under the tested mating conditions and remained detectable during serial passage, whereas KPC-287 alone did not measurably enhance virulence in the ATCC 13883 background. These findings identify KPC-287 as a potentially clinically relevant CAZ/AVI resistance determinant and underscore the importance of genomic surveillance and functional characterization of emerging KPC variants.

## Data Availability

The datasets presented in this study can be found in online repositories. The names of the repository/repositories and accession number(s) can be found in the article/Supplementary material.

## Footnotes

1
https://itol.embl.de/

2
https://www.genome.jp/tools-bin/clustalw

3
http://espript.ibcp.fr/ESPript/

4
https://alphafoldserver.com/
