# Supplementary material for: Characterization of KPC-287 conferring high-level ceftazidime–avibactam resistance in an ST11-KL64 Klebsiella pneumoniae isolate
Source: Front Microbiol. 2026 Oct 16;17:1938185. doi: 10.3389/fmicb.2026.1938185 (PMC13719599; doi:10.3389/fmicb.2026.1938185)
Supplement: Supplementary file 1 [file Data_Sheet_1.DOCX]

**Figure S1. Nucleotide sequence comparison of *bla*_KPC-287_ and *bla*_KPC-155_.**

Nucleotide sequences of KPC-155 and KPC-287 variant were aligned using ClustalW and presented using ESPript 3.0.
