# Supplementary material for: Characterization of KPC-287 conferring high-level ceftazidime–avibactam resistance in an ST11-KL64 Klebsiella pneumoniae isolate
Source: Front Microbiol. 2026 Oct 16;17:1938185. doi: 10.3389/fmicb.2026.1938185 (PMC13719599; doi:10.3389/fmicb.2026.1938185)
Supplement: Supplementary file 2 [file Table_1.DOCX]

**Table S1. Primers used in this study.**

| **Target** | **Primer name** | **Sequence （5' - 3')** |
| --- | --- | --- |
| pUC19 | pUC19-rPCR-F | GAATTCACTGGCCGTCGTTT |
|  | pUC19-rPCR-R | AAGCTTGGCGTAATCATGGTCA |
| *bla*_KPC-287_ of KP22-83 | KPC-287-F | accatgattacgccaagcttGCAGACCAACGGCATGGTG |
|  | KPC-287-R | aaacgacggccagtgaattcTTACTGCCCGTTGACGCC |
